# A Population Based Study of Liver Function amongst Adults with Hyperuricemia and Gout in the United States

**DOI:** 10.3390/diseases9030061

**Published:** 2021-09-17

**Authors:** Subrata Deb, Prashant Sakharkar

**Affiliations:** 1 Department of Pharmaceutical Sciences, College of Pharmacy, Larkin University, Miami, FL 33169, USA; sdeb@alumni.ubc.ca; 2 Clinical and Administrative Sciences, Roosevelt University College of Science, Health and Pharmacy, Schaumburg, IL 60173, USA

**Keywords:** hyperuricemia, gout, NHANES, liver dysfunction, association

## Abstract

To examine the association between uric acid levels and liver enzyme functions amongst adults with hyperuricemia and gout in the United States. The National Health and Nutrition Examination Survey (NHANES) data from 2007 to 2016 was used to study the research objective. Data were analyzed for descriptive statistics and for differences using the t test, Chi-square test and ANOVA. A regression analysis was performed to determine association between demographics and liver enzymes. A *p* value of <0.05 or <0.001 was considered statistically significant. A total of 14,946 adults (≥20 yrs.) were included in this study. Sample mean age was 49 ± 0.15 yrs., and 54% were female. Overall, 15% adults had elevated uric acid levels (≥6.8 mg/dL), men had significantly higher uric acid levels than women (6 mg/dL vs. 4.8 mg/dL). High uric acid levels were associated with more than two times higher odds of elevated ALT, AST and GGT (*p* < 0.001). Similarly, gender-based target uric acid values were associated with two-fold increased odds of GGT, over one-and-a-half fold higher odds of ALT and AST (*p* < 0.001). Regression analysis showed significant association between age, gender, race/ethnicity, body mass index, and hypertension and ALT, AST, ALP, total bilirubin and GGT (*p* < 0.001). Adults with hyperuricemia and gout are most likely to develop liver dysfunctions and suffer associated morbidities. Such patients need to be appropriately monitored and managed for their liver functions and to prevent associated morbidities.

## 1. Introduction

The prevalence of hyperuricemia and gout in US population is approximately 20% and 4%, respectively [1]. According to Chen-Xu et al. (2019), a staggering 47.1 million adults were estimated to be diagnosed with hyperuricemia considering gender-specific uric acid levels in 2015–2016 [1]. Though there is considerable variability in the definition of hyperuricemia, uric acid levels >6.8 mg/dL is widely accepted as hyperuricemia in the general population [2]. However, there are gender-specific differences in the hyperuricemia criteria with serum urate levels of ≥6.8 mg/dL for men and ≥5.7 mg/dL for women designated as elevated levels [1]. Hyperuricemia or elevated levels of uric acid is a metabolic disorder that is known to be a major precursor for an inflammatory condition called gout. The formation of urate crystals from elevated uric acid levels and subsequent precipitation of those crystals trigger development of gout [3]. Although not all adults with hyperuricemia experience gout-related symptoms and progression. The inflammatory arthritis which is primarily caused by hyperuricemia is the primary symptoms of gout. Several comorbidities including overweight, hyperglycemia, elevated blood pressure and renal disorder contribute to hyperuricemia [3]. About 70% of the uric acid is renally excreted and thus certain drugs and increased concentrations of lead, lactates and ketones can block the renal excretion of uric acid, leading to accumulation of serum uric acid and development of hyperuricemia [4].

Liver function is critical for biosynthesis/metabolism of endogenous compounds as well as elimination of xenobiotics. Hepatic stress from endogenous or exogenous substances initiates liver cell growth which can stimulate liver enzymes such as alanine aminotransferases (ALT), aspartate aminotransferases (AST) and alkaline phosphatase (ALP) [5]. For uric acid biosynthesis, liver is the primary site with the highest protein expression of xanthine oxidase which is the main enzyme responsible for uric acid formation [6]. Thus, hepatocytes are consistently exposed to uric acid at a very high level. Cell culture studies with hepatocytes suggest that uric acid has the capability to cause mitochondrial oxidative stress and thus potentially trigger liver dysfunction [7]. Despite a high number of individuals experiencing hyperuricemia, there is limited information available about the effect of hyperuricemia on liver function in population-based observational studies. The goal of the present population-based cross-sectional study was to analyze liver function amongst adults with hyperuricemia and gout in the United States.

## 2. Methods

### 2.1. Study Population

We analyzed the sample of adults 20 years and older who participated in the National Health and Nutrition Examination Survey (NHANES), an ongoing population-based statistical survey designed to assess the health and nutritional status of adults and children in the US. NHANES uses a representative sample of the noninstitutionalized US civilian population that is selected using a multistage, stratified sampling design. The survey is unique in that it combines interviews, physical examinations and various laboratory data. In-person interviews were conducted in sampled households, and subjects were invited to participate in medical examinations. We extracted data on individuals who participated in NHANES from 2007 through 2016 into a combined dataset (NHANES 2007–2016) to increase sample size for greater estimator reliability (NHANES Analytic Guidelines) [8]. Of the total 51,694 participants on whom information was gathered during interview, 48,710 (94%) were also screened for laboratory data. We excluded individuals (26,983) who either had hepatitis B surface antibody (*n* = 12,599), hepatitis B surface antigen (159), hepatitis D antibody (43) and hepatitis C confirmed antibody combined (*n* = 336), and hepatitis A antibody (21,737) and hepatitis B core antibody (*n* = 2071). We also excluded participants who self-reported of having liver condition on the interview (1127) and with either missing AST, ALT, ALP, GGT, total bilirubin and uric acid levels (*n* = 19,797) leaving final adjusted sample of 14,946 participants of age 20 years old or above for the analyses.

### 2.2. Covariates

Covariates included age, gender, ethnicity/race, education, income, poverty level, Body Mass Index (BMI), Systolic Blood Pressure (SBP), Diastolic Blood Pressure (DBP). Age, ethnicity/race, income, knowledge of having gout was self-reported by the participants.

### 2.3. Assessment of Gout and Hyperuricemia

Timed endpoint method was used to measure the concentration of uric acid in serum, plasma, or urine. Uric acid is oxidized by uricase to produce allatoin and hydrogen peroxide. The hydrogen peroxide reacts with 4-aminoantipyrine (4-AAP) and 3,5-dichloro-2-hydroxybenzene sulfonate (DCHBS) in a reaction catalyzed by peroxidase to produce a colored product. The system monitors the change in absorbance at 520 nm at a fixed time interval. The change in absorbance is directly proportional to the concentration of uric acid in the sample. Details of quality-control procedures have been published elsewhere (http://www.cdc.gov/nchs/data/nhanes/nhanes3/cdrom/nchs/manuals/labman.pdf, accessed on 10 May 2021).

Our primary definition of gout and hyperuricemia was in accordance with the American College of Rheumatology 2020 Guidelines for the uric acid range [2]. Individuals were considered of having hyperuricemia if they either had uric acid level of 6.8 mg/dL, which is the standard target level, or having gout if they answered yes to the question “Doctor ever told you that you had gout” in the home interview. We also examined the potential impact of alternative definitions of hyperuricemia that included gender-based distinction of serum uric acid level ≥6.8 mg/dL in male and ≥5.7 mg/dL in female. Uric acid values reported in mg/dL can be converted to μmoles/liter by multiplying by 59.48. Body mass index, a measure of obesity defined as weight in kilograms divided by height in meters squared, was categorized according to clinical guidelines set by the National Institute of Health [9] and hypertension was defined as blood pressure of 130/85 mmHg according to the National Cholesterol Education Program-Adult Treatment Panel-III (NCEP-ATP III) guidelines [10].

### 2.4. Statistical Analysis

The statistical analyses for this study were performed using STATA ver14 (STATA Corp, College Station, TX, USA) a statistical software package that considers sample weighting and the complex, multistage probability sample design of NHANES [11]. Demographic characteristics were compared by age, gender, race/ethnicity, education and uric acid levels using the Chi-square test followed by post-hoc analyses with Bonferroni correction. Sampling weights were applied to account for selection probabilities, oversampling, nonresponse, and differences between the sample and the US adolescent male population. We examined the association between uric acid and liver enzyme levels. Logistic regression analyses were performed to quantify the magnitude of associations between the universal uric acid levels and gender-based uric acid levels and liver enzyme levels in presence of covariates. There is no universal agreement about the cut-off values for ALT and AST in published literature. In this study, we defined abnormal ALT and AST values as a range (<20 mg/dL, 20–29 mg/dL, 30–39 mg/dL and ≥40 mg/dL) for total sample population. A threshold value of 17 mmol/dL for total bilirubin, 40 U/L for GGT and AST/ALT ratio of 1 was used. We also used a gender-based target value of ALT (≥30 U/L for male and ≥25 U/L for female) and AST (≥33 U/L for male and female both), and GGT (≥65 IU/L for male, ≥36 IU/L for female) to explore if this change in target value produces any difference in associations [12]. These target values were chosen as they represent common institutional reference values and generally used in the clinical practice. Similar target values have been used in both adolescent and adult in earlier epidemiological studies [13,14]. Regression model was adjusted for age, gender, ethnicity, BMI, hypertension and uric acid level. Taylor series linearization was used for variance estimation. A *p* value of ≤0.05 or ≤0.001 was considered statistically and highly statistically significant, respectively.

## 3. Results

### 3.1. Study Participants

Table 1 presents the demographic characteristics of individuals with gout and hyperuricemia. A total of 14,946 adults (20 years and above) were included in this study. The mean age of the sample population was 49 ± 0.15 yrs. and 52% individuals were between age 20 and 40 years, 54% were female, 55% were non-Hispanic White, and 60% had some college or graduate level education. A total of 60% individuals had family income of less than USD 55,000, 67% were overweight and obese, 27% had hypertension and 84% were taking prescription medication for their hypertension (Table 1). The mean BMI was 31 kg/m^2^ and waist circumference was 106.1 cm in individuals with hyperuricemia, above the target value of 30 for BMI and waist circumference of 102 cm for men, and 88 cm for women (Table 1). Fifteen percent of adults had uric acid level 6.8 mg/dL in overall sample. Men had significantly higher serum uric acid levels than women (6 mg/dL vs. 4.8 mg/dL). Mean serum uric acid level in overall sample among adults was 5.4 mg/dL, whereas it was 7.8 mg/dL in hypouricemic individuals, quite higher than the threshold value of 6.8 mg/dL recommended by the American Rheumatology Association (Table 1).

### 3.2. Association of Participants Characteristics and Uric Acid Levels

We examined the association of demographic characteristics with serum uric acid level using the Chi-square test. High serum uric acid levels were significantly associated with age, gender, race/ethnicity, education and family income. Individuals of age 45 years and above and being male showed significant association with higher serum uric acid levels. In general, among both men and women, the higher uric acid levels were associated with an increasing blood pressure and body mass index. Individuals with history of hypertension had a significantly higher serum uric acid level than those who did not. Among individuals with gout and hyperuricemia, 76% were male, 54% were non-Hispanic White, 61% had hypertension and 85% were on prescription medication for hypertension and 53% were obese. These proportions were substantially and significantly higher than those among individuals without gout and hyperuricemia (Table 1).

### 3.3. Association of Uric Acid Levels with Abnormal Liver Enzymes

We also examined the association of serum uric acid levels with liver enzymes. Uric acid levels were found to be significantly associated with liver enzymes ALT and AST, AST/ALT ratio and total bilirubin (Table 2). Post hoc analysis showed high uric acid level was significantly associated with high ALT (≥30), AST (≥33) and total bilirubin levels (≥17) except for ALP. Forty percent of individuals had high ALT (≥30), 31% had high AST (≥33) and 23% had high total bilirubin levels (≥17) (Table 2). High uric acid levels were also found significantly associated with gender-based target values for ALT ≥30 U/L in male and ≥25 U/L in female, 33 U/L in both male and female for AST and ≥65 U/L in male and ≥36 U/L in female for GGT compared to normal uric acid levels. Thirteen percent of individuals had high ALT, 22% had high AST and 42% had high total bilirubin levels (Table 3).

We also calculated the odds ratio of abnormal ALT, AST, AST/ALT ratio, ALP, GGT and total bilirubin using both overall and gender-based target value of serum uric acid. Uric acid levels were associated with over two times higher odds of elevated ALT (OR: 2.22, 95%CI: 1.94, 2.54, *p* < 0.001), AST (OR: 2.36, 95% CI: 2.02, 2.77, *p* < 0.001), GGT (OR: 2.20, 95% CI: 1.88, 2.57, *p* < 0.001) and over one-and-a-half times of higher odds of total bilirubin (OR: 1.89, 95% CI: 1.64, 2.18, *p* < 0.001) (Table 4). Gender-based target values of uric acid were associated with two-fold increased odds of GGT (OR: 2.26, 95%CI: 1.90, 2.69, *p* < 0.001), over one and half fold higher odds of ALT (OR: 1.90, 95%CI: 1.70, 2.11, *p* < 0.001), AST (OR:1.84, 95%CI: 1.59, 2.13, *p* < 0.001) whereas, over one times higher odds of total bilirubin (OR:1.29, 95%CI: 1.13, 1.47, *p* < 0.001) (Table 4).

### 3.4. Association of Gender-Based Target Values of Uric Acid with Abnormal Liver Enzymes

Gender based target values of serum uric acid levels were also found significantly associated with ALT, AST, AST/ALT ratio and total bilirubin. Gender-based target values of serum uric acid were also found to be associated and gender-based target values of ALT (≥30 U/L in male and ≥25 U/L in female), AST (33 U/L in both male and female) and GGT (≥65 U/L in male and ≥36 U/L in female).

### 3.5. Predictors of High Liver Enzymes Levels on Regression Analyses

We modeled the odds of abnormal ALT (≥30 U/L for male and ≥25 U/L for female) and AST (≥33 U/L for male and female both), AST/ALT ratio (≥1), ALP (≥120 U/L), GGT (≥65 IU/L for male, ≥36 IU/L for female) and total bilirubin (≥17 mmol/dl) using uric acid and other covariates simultaneously and calculated the odds ratio using a logistic regression model (Table 5). On regression analysis, after adjustment, serum uric acid level was associated with higher ALT (OR:1.57, 95%CI: 1.36, 1.80, *p* < 0.001), AST (OR:1.68, 95%CI: 1.42, 2.00, *p* < 0.001), GGT (OR:1.86, 95%CI: 1.47, 2.34, *p* < 0.001) and total bilirubin (OR:1.79, 95%CI: 1.51, 2.12, *p* < 0.001). BMI was associated with higher ALT (OR:1.06, 95%CI: 1.05, 1.07, *p* < 0.001), AST (OR:1.02, 95%CI: 1.01, 1.03, *p* < 0.001), ALP (OR:1.04, 95%CI: 1.02, 1.05, *p* < 0.001) and GGT levels (OR:1.04, 95%CI: 1.03, 1.05, *p* < 0.001). Hypertension was also associated with higher ALT (OR:1.19, 95%CI: 1.03, 1.36, *p* = 0.015), AST (OR:1.27, 95%CI: 1.08, 1.50, *p* = 0.005), ALP (OR:1.38, 95%CI: 1.04, 1.84, *p* = 0.028) and GGT (OR:1.43, 95%CI: 1.18, 1.72, *p* < 0.001). Age was also associated with high ALP (OR:1.02, 95%CI: 1.00, 1.03, *p* < 0.001) and GGT (OR:1.01, 95%CI: 1.00, 1.01, *p* < 0.001). Female gender was associated with lower ALT (OR:0.50, 95%CI: 0.98, 0.99, *p* < 0.001), AST (OR:0.45, 95%CI: 0.40, 0.50, *p* < 0.001) and total bilirubin (OR:0.37, 95%CI: 0.37, 0.43, *p* < 0.001), whereas with high GGT levels (OR:1.89, 95%CI: 1.56, 2.29, *p* < 0.001). Race/ethnicity was associated with low ALT (OR:0.86, 95%CI: 0.81, 0.90, *p* < 0.001), AST (OR:0.92, 95%CI: 0.87, 0.96, *p* < 0.001) and ALP (OR:0.80, 95%CI: 0.72, 0.89, *p* < 0.001). Other Hispanics had lower ALT, AST and ALP levels compared to another race/ethnicity (Table 5).

Similar pattern was observed when gender-based target serum uric acid level was used (Supplemental Figures S1 and S2). After adjustment, uric acid level was associated with higher ALT (OR:1.58, 95%CI: 1.40, 1.77, *p* < 0.001), AST (OR:1.60, 95%CI: 1.37, 1.87, *p* < 0.001), GGT (OR:1.81, 95%CI: 1.52, 2.17, *p* < 0.001) and total bilirubin (OR:1.63, 95%CI: 1.42, 1.88, *p* < 0.001) Race/ethnicity was associated with low ALT (OR:0.82, 95%CI: 0.81, 0.89, *p* < 0.001), AST (OR:0.91, 95%CI: 0.87, 0.96, *p* < 0.001) and ALP (OR:0.81, 95%CI: 0.73, 0.90, *p* < 0.001). Other Hispanics had lower ALT, AST and ALP levels compared to another race/ethnicity (Supplemental Table S1).

## 4. Discussion

Hyperuricemia is the primary cause of a condition called gout. In addition to inflammatory effects, uric acid can cause cellular toxicity [15]. In the present study, the participants from 2007 to 2016 NHANES study cycles were evaluated for availability of information on gout, uric acid level and liver enzyme function indicators. The main objective of this study was to examine any association among the uric acid levels and liver function as determined by AST, ALT and ALP in 14,946 adults with serum uric acid level and individuals with reported diagnosis of gout. Demographic characteristics indicate that the study population has a mean age of 49.3 with slightly over 50% of the study subjects were in the age range of 20 to 44 years with approximately equal representation of each sex in the sample. The results from our analyses suggest that adults ≥49-years-old and being male had elevated serum uric acid levels. In general, majority of the participants had increased blood pressure, were overweight, and higher BMI. Similar to other metabolic disorders, hyperuricemia was also found to be associated with race/ethnicity, education, and family income.

Though there are gender-specific differences in the cut-off values for hyperuricemia, ≥6.8 mg/dL is considered a reasonable clinical target to avoid any gout-related complications [2]. In our NHANES samples, the mean uric acid levels were 5.4 mg/dL for the whole study population with mean level of 5.0 mg/dL among the group categorized as <6.8 mg/dL and 7.8 mg/dL mean uric acid level in the group categorized as ≥6.8 mg/dL. High liver function markers (AST ≥33, ALT ≥30, total bilirubin ≥17) were significantly associated with high uric acid levels (≥6.8 mg/dL). The gender-based cut-off values for upper normal levels of AST, ALT and GGT was significantly associated with serum uric acid levels. The individuals with elevated uric acid levels had two times higher odds of abnormal AST, ALT, or GGT functions. Similarly, gender-based target values of uric acid indicated higher odds of getting diagnosed with elevated liver functions among the participants. Regression models suggest that uric acid is a good predictor of abnormal AST, ALT, GGT and total bilirubin levels. Similarly, age, gender, race and BMI covariates were also strongly associated with higher liver functions. Gender-based cut-off values of uric acid mirrored the results from the overall value of elevated uric acid (≥6.8 mg/dL).

Previous population studies with subjects from earlier timelines or from different countries have highlighted the relationship between serum uric acid levels and a variety of liver dysfunction. Based on NHANES data from 1988–1994 and 1999–2006, Afzali and colleagues concluded that elevated uric acid levels pose a higher risk of developing liver cirrhosis and its related severity [16]. Likewise, analysis of 1988–1994 NHANES data in another study indicated that increased levels of uric acid was connected to non-alcoholic fatty liver disease (NAFLD) that was confirmed on ultrasound screening [17]. Interestingly, the cohort of subjects included in the study were nondiabetic, thus eliminating a major contributing factor to NAFLD [14] and highlighting the independent role of uric acid in NAFLD. In a cross-sectional study in China, elevated uric acid and obesity synergistically influenced the development and progression of NAFLD as determined by ultrasound screening [18]. Similarly, Shih et al., (2015) in their research reiterated the relationship between serum uric acid and NAFLD in US population [19]. In another study conducted among US pediatric patients, serum, uric acid levels were associated with nonalcoholic steatohepatitis potentially mirroring fructose intake by the children [20]. Catanzaro et al. (2020) reported an association between elevated uric acid and NAFLD in European Mediterranean individuals [21]. Incidentally, there have been quite a few cross-sectional and prospective observational studies with Chinese population analyzing serum uric acid and liver health. Bao et al. (2020) in their study identified a relationship between serum uric acid level and NAFLD in a population of non-obese post-menopausal women [22]. Similarly, in another prospective observational study, individuals with elevated serum uric acid levels were found to have a greater potential to develop NAFLD [23]. Several other population studies reported from Japan, Greece, Turkey, Korea and Israel indicated elevated serum uric acid as a potential risk factor for developing some form of liver dysfunction [24,25,26,27,28].

**Figure 1:**
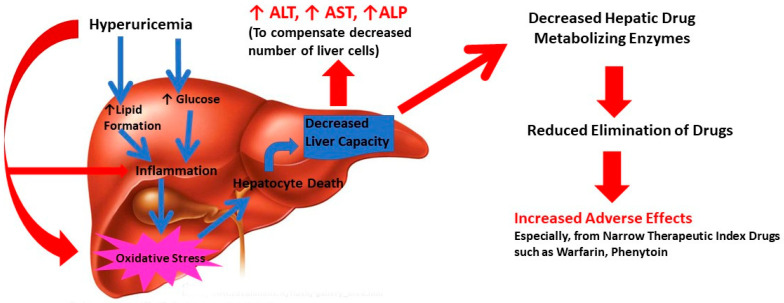
Potential pathophysiological mechanism of hyperuricemia related liver dysfunction in gout.

Liver dysfunction can result directly through elevated uric acid level through multi-dimensional mechanisms. It can induce inflammation and oxidative stress, leading to liver cell death and decrease in functional hepatocytes through lipids and glucose metabolism [7]. Interestingly, uric acid has the potential to cause oxidative stress and mitochondrial dysfunction directly [7]. Uric can also increase lipid synthesis by stimulating endoplasmic reticulum (ER) stress and though Sterol regulatory element-binding protein 1 (SREBP-1) and other transcriptional elements [29]. Increased lipid levels in turn can cause inflammation and oxidative stress. In addition, uric acid can promote cellular glucose biosynthesis which can catalyze increased inflammation in liver [6]. Thus, uric acid either directly or via lipids and glucose can induce inflammation and oxidative stress, leading to liver cell death and decrease in functional hepatocytes (Figure 1). These events reduce the liver’s functional capacity resulting in elevated levels of liver enzyme functions (ALT, AST, ALP). As a secondary outcome of compromised liver function, the liver drug metabolizing capacity and ability to eliminate drugs can decrease. This can reflect in increased drug adverse effects and potential treatment failure [30]. It is worth recognizing that hyperuricemia is the driving force behind development of gout, a severe inflammatory condition [3]. Indeed, levels of several inflammatory markers including white blood cell, neutrophil count, C-reactive protein, interleukin-6, interleukin-18 and tumor necrosis factor-α were higher in hypouricemic conditions [31]. The cellular and tissue alterations of liver are considered directly responsible for histopathogenesis of NAFLD including steatosis and non-alcoholic steatohepatitis [32,33]. In addition, recent reports suggest that hyperuricemia is a potential marker for cardio-metabolic disease including arrythmia [34,35]. Thus, the mechanism of uric acid-driven liver dysfunction can be complex and multifaceted and appears to occur through inflammation-related pathways. The results from the current study suggest that elevated serum uric acid levels are associated with abnormal liver function.

Our study has several limitations even though it was performed using the current nationally representative sample of noninstitutionalized US civilian population. This study was not able to establish potential temporal relations between hyperuricemia and abnormal liver enzymes, which can be further addressed by longitudinal prospective studies. The diagnosis of gout by a physician among the participants was self-reported and could not be validated. However, it is unlikely that such misclassification would have provided such strong associations observed in this population study. We used liver enzyme levels only as a surrogate marker in the absence of liver ultrasound examination to assess the hepatic dysfunction. Furthermore, the serum uric acid levels in this study were ascertained objectively without reliance on participants’ recall and showed consistent results.

## 5. Conclusions

Individuals with hyperuricemia and gout were at one to two times higher risk of having elevated liver enzyme levels. Men had significantly higher uric acid levels than women. Female had lower ALT, AST and total bilirubin, whereas they had higher GGT levels compared to men. Other Hispanics had lower ALT, AST and ALP levels compared to another race/ethnicity. Adults with hyperuricemia and gout need to be appropriately monitored and managed for their liver functions and to prevent associated morbidities.

## Figures and Tables

**Table 1:** Demographic Characteristics and Laboratory Data of NHANES participants (2007–2016).

		Uric Acid		
	Total Sample N = 14,946	Normal *n* = 12,714 (85.0)	Elevated *n* = 2232 (15.0)	*p*-Value
Age (yrs.) (Mean ± SE)	49.3 (0.15)	48.2 (0.16)	51.4 (0.40)	<0.001 **
**Age (yrs.)**				
20–44	6447 (51.9)	5712 (52.9)	735 (45.5)	<0.001 **
45–64	4771 (29.2)	4080 (29.3)	691 (28.3)	
≥65	3728 (18.9)	2922 (17.8)	806 (26.2)	
**Gender**				
Male	7037 (45.8)	5539 (44.1)	1678 (76.2)	<0.001 **
Female	7909 (54.2)	7355 (58.9)	554 (23.8)	
**Marital Status**				
Single	2820 (21.5)	2447 (21.5)	373 (21.2)	0.960
Married	7664 (52.6)	6490 (52.5)	1174 (52.9)	
Other	4456 (25.9)	3771 (26.0)	685 (25.9)	
**Race/Ethnicity**				
Mexican American	3300 (14.2)	2916 (14.5)	384 (12.2)	<0.001 **
Other Hispanic	2105 (8.7)	1856 (8.8)	249 (7.3)	
Non-Hispanic White	4324 (55.3)	3637 (53.2)	687 (54.4)	
Non-Hispanic black	3020 (12.3)	2428 (11.9)	592 (15.1)	
Other	2197 (11.5)	1877 (11.6)	320 (11.0)	
**Education**				
<9th Grade	2087 (9.6)	2087 (9.6)	384 (11.2)	<0.001 **
9–11th Grade	1935 (12.0)	1935 (12.0)	364 (12.8)	
High School/GED	2565 (19.0)	2565 (19.0)	515 (24.3)	
Some College or AA degree	3305 (29.9)	3305 (29.9)	556 (28.8)	
College Graduate or above	2808 (29.5)	2808 (29.5)	409 (22.9)	
**Family Income**				
<USD 25,000	5078 (28.4)	4270 (28.2)	808 (29.9)	0.020 *
USD 25,000–USD 54,999	4690 (31.6)	3997 (31.4)	693 (32.8)	
USD 55,000–USD 99,999	2445 (20.1)	2074 (19.9)	371 (20.9)	
>USD 100,000	1882 (19.9)	1652 (20.4)	230 (16.4)	
**Ratio of Family Income to Poverty**				
<1.35	5108 (28.6)	4357 (28.6)	751 (28.4)	0.027 *
1.35–1.84	1717 (10.8)	1440 (10.7)	277 (11.6)	
1.85–2.99	2401 (17.9)	2021 (17.5)	380 (20.3)	
≥3.00	4219 (42.7)	3626 (43.2)	593 (39.7)	
**BMI (kg/m^2^)**				
Underweight	240 (1.8)	229 (2.0)	11 (0.5)	<0.001 **
Normal	4301 (31.5)	3972 (34.2)	329 (14.3)	
Overweight	5021 (33.1)	4306 (33.2)	715 (32.6)	
Obese	5210 (33.6)	4073 (30.6)	1137 (52.6)	
**SBP**				
<135 mmHg	9903 (75.1)	8642 (76.7)	1261 (65.2)	<0.001 **
≥135 mmHg	4474 (24.9)	3577(25.3)	897 (34.8)	
**DBP**				
<85 mmHg	13,186 (92.7)	11,303 (93.6)	1883 (87.1)	<0.001 **
≥85 mmHg	1191 (7.3)	916 (6.4)	275 (12.9)	
**Hypertension (135/85 mmHg)**				
Yes	9633 (73.2)	8834 (75.0)	1199 (61.9)	<0.001 **
No	4744 (26.8)	3785 (25.0)	959 (38.1)	
**Taking prescription for hypertension**				
Yes	4447 (84.1)	3388 (83.6)	1059 (85.9)	0.208
No	622 (15.9)	511 (16.4)	111 (14.1)	
BMI (kg/m^2^) (Mean ± SE)	28.6 (0.05)	28.1 (0.06)	31.1 (0.14)	<0.001 **
Waist Circumference (cm) (Mean ±SE)	97.5 (0.13)	96 (0.14)	106.1 (0.34)	<0.001 **
SBP (mmHg) (Mean ± SE)	123.8 (0.2)	122.9 (0.17)	129.0 (0.42)	<0.001 **
DBP (mmHg) (Mean ± SE)	69.1 (0.11)	68.9 (0.11)	70.1 (0.32)	<0.001 **
Uric Acid (mg/dL) (Mean ± SE)	5.4 (0.01)	5.0 (0.01)	7.8 (0.02)	<0.001 **

Data presented as frequency and percent in parentheses unless specified; Normal uric acid level (<6.8 mg/dL); Elevated uric acid level (≥6.8 mg/dL); BMI: Body Mass Index; SBP: Systolic blood pressure; DBP: Diastolic blood pressure; Significant at * *p* < 0.05 and ** *p* < 0.001.

**Table 2:** Association between Hyperuricemia and Liver Enzymes.

	**ALT (U/L)**					**AST/ALT Ratio**			**Total Bilrubin (μmol/L)**		
**Uric Acid**	**<20**	**≥20–29**	**≥30–39**	**≥40**	***p*-Value**	**<1**	**≥1**	***p*-Value**	**<17**	**≥17**	***p*-Value**
Normal	6056 (48.4)	4203 (32.4) ^a^	1305 (10.5)	1150 (8.7)	<0.001 **	4035 (35.6)	8679 (68.4)	<0.001 **	11,078 (86.7)	1636 (13.3)	<0.001 **
Elevated	702 (25.4)	789 (35.2) ^a^	368 (19.2)	373 (20.2)		939 (48.7)	1293 (51.3)		1800 (77.5)	432 (22.5)	
	**AST (U/L)**					**ALP (U/L)**					
	**<20**	**≥20–29**	**≥30–39**	**≥40**		**<120**	**≥120**				
Normal	3332 (26.0)	7225 (56.7) ^a^	12,362 (98.0)	352 (2.0)	<0.001 **	12,362 (98.0)	352 (2.0)	0.541			
Elevated	332 (11.8)	1268 (57.5) ^a^	2164 (97.8)	68 (2.2)		2164 (97.8)	68 (2.2)				

Data presented as frequency and percent in parentheses. Normal uric acid level (<6.8 mg/dL); Elevated uric acid level (≥6.8 mg/dL); Significant at ** *p* < 0.001. ^a^ Subscript letter denotes category whose column proportions did not differ significantly from others on post hoc analyses with Bonferroni correction.

**Table 3:** Association between Hyperuricemia and Liver Enzymes (Gender-based cut-off value).

	**ALT (U/L)**		
**Uric Acid (mg/dL)**	**Normal**	**Elevated**	***p*-Value**
Normal	11,562 (92.5)	1152 (7.5)	<0.001 **
Elevated	1942 (87.2)	290 (12.8)	
**Uric Acid (mg/dL)**	**AST (U/L)**		
	**Normal**	**Elevated**	
Normal	11,276 (89.4)	1438 (10.6)	<0.001 **
Elevated	1785 (78.1)	447 (21.9)	
**Uric Acid (mg/dL)**	**GGT (U/L)**		
	**Normal**	**Elevated**	
Normal	9579 (75.8)	3135 (24.2)	<0.001 **
Elevated	1437 (58.5)	795 (41.5)	

Data presented as frequency and percent in parentheses; Normal uric acid level (<6.8 mg/dL); Elevated uric acid level (≥6.8 mg/dL); Normal ALT (<30 U/L for Male and <25 U/L for Female); Elevated ALT (≥30 U/L for Male and ≥25 U/L for Female); Normal AST (<33 U/L for both Male and Female); Elevated AST (≥33 U/L for both Male and Female); Normal GGT (<65 U/L for Male and <36 U/L for Female); Elevated GGT (≥65 U/L for Male and ≥36 U/L for Female); Significant at ** *p* < 0.001.

**Table 4:** Odds of Elevated Liver Enzymes with Uric acid (Universal and gender-based cut-off value).

**Uric Acid (mg/dL)**	**ALT (U/L)**		**AST (U/L)**		**AST/ALT Ratio**	
	**OR (95%CI)**	***p*-Value**	**OR (95%CI)**	***p*-Value**	**OR (95%CI)**	***p*-Value**
Universal	2.22 (1.94, 2.54)	<0.001 **	2.36 (2.02, 2.77)	<0.001**	0.49 (0.42, 0.56)	<0.001 **
Gender-based	1.90 (1.70, 2.11)	<0.001 **	1.84 (1.59, 2.13)	<0.001**	0.64 (0.58, 0.72)	<0.001 **
	**ALP (U/L)**		**GGT (U/L)**		**Total Bilirubin (μmol/L)**	
**Uric Acid (mg/dL)**	**OR (95%CI)**	***p*-Value**	**OR (95%CI)**	***p*-Value**	**OR (95%CI)**	***p*-Value**
Universal	1.11 (0.80, 1.53)	0.542	2.20 (1.88, 2.57)	<0.001 **	1.89 (1.64, 2.18)	<0.001 **
Gender-based	1.09 (0.82, 1.47)	0.539	2.26 (1.90, 2.69)	<0.001 **	1.29 (1.13, 1.47)	<0.001 **

Elevated universal (≥6.8 mg/dL); Elevated gender-based (≥6.8 mg/dL in male and ≥5.7 mg/dL in female); OR = Odds Ratio; CI = Confidence Interval; Significant at ** *p* < 0.001.

**Table 5:** Predictors of Liver Enzymes on Regression (Adjusted Model).

	**ALT (U/L)**		**AST (U/L)**		**AST/ALT Ratio**	
	**OR (95%CI)**	***p*-Value**	**OR (95%CI)**	***p*-Value**	**OR (95%CI)**	***p*-Value**
Age	0.99 (0.98, 0.99)	<0.001 **	0.99 (0.99, 1.00)	0.365	1.02 (1.01, 1.02)	<0.001 **
Gender	0.50 (0.45, 0.56)	<0.001 **	0.45 (0.40, 0.50)	<0.001 **	3.58 (3.20, 4.00)	<0.001 **
Race	0.86 (0.81, 0.90)	<0.001 **	0.92 (0.87, 0.96)	<0.001 **	1.18 (1.13, 1.23)	<0.001 **
BMI	1.06 (1.05, 1.07)	<0.001 **	1.02 (1.01, 1.03)	<0.001 *	0.92 (0.91, 0.93)	<0.001 **
HTN	1.19 (1.03, 1.36)	0.015 *	1.27 (1.08, 1.50)	0.005 *	0.95 (0.84, 1.06)	0.348
Uric Acid	1.57 (1.36, 1.80)	<0.001 **	1.68 (1.42, 2.00)	<0.001 **	0.84 (0.71, 0.98)	0.029 *
	**ALP (U/L)**		**GGT (U/L)**		**Total Bilirubin (μmol/L)**	
	**OR (95%CI)**	***p*-Value**	**OR (95%CI)**	***p*-Value**	**OR (95%CI)**	***p*-Value**
Age	1.02 (1.00, 1.03)	<0.001 **	1.01 (1.00, 1.01)	<0.001 **	0.99 (0.99, 1.02)	0.382
Gender	1.34 (0.96, 1.88)	0.082	1.89 (1.56, 2.29)	<0.001 **	0.37 (0.32, 0.43)	<0.001 **
Race	0.80 (0.72, 0.89)	<0.001 **	0.96 (0.89, 1.03)	0.264	0.99 (0.93, 1.05)	0.650
BMI	1.04 (1.02, 1.05)	<0.001 **	1.04 (1.03, 1.05)	<0.001 **	0.93 (0.92, 0.94)	<0.001 **
HTN	1.38 (1.04, 1.84)	0.028 *	1.43 (1.18, 1.72)	<0.001 **	0.96 (0.81, 1.13)	0.608
Uric Acid	1.00 (0.66, 1.53)	0.967	1.86 (1.47, 2.34)	<0.001 **	1.79 (1.51, 2.12)	<0.001 **

OR = Odds Ratio; CI = Confidence Interval; BMI: Body Mass Index; HTN: Hypertension (135/85 mmHg); Significant at * *p* < 0.05 and ** *p* < 0.001.

## Data Availability

NHANES data is available in a publicly accessible repository through Centers for Disease Control and Prevention (CDC) and the National Center for Health Statistics (NCHS). The data presented in this study are openly available in the National Health and Nutrition Examination Survey (NHANES) website at https://wwwn.cdc.gov/nchs/nhanes/ (accessed on 14 July 2021).

## Supplementary Materials

The following are available online at https://www.mdpi.com/article/10.3390/diseases9030061/s1, Figure S1: Forest plots of the odds of elevated liver enzymes with hyperuricemia using universal cut-off value; Figure S2: Forest plots of the odds of elevated liver enzymes with hyperuricemia using gender-based cut-off values; Table S1: Predictors of Liver Enzymes on Regression (Adjusted Model).
