# Supplementary material for: A Population Based Study of Liver Function amongst Adults with Hyperuricemia and Gout in the United States
Source: Diseases. 2021 Sep 17;9(3):61. doi: 10.3390/diseases9030061 (PMC8482250; doi:10.3390/diseases9030061)
Supplement: Supplementary file 1 [file diseases-09-00061-s001.zip › diseases-1320074-supplementary.pdf]

diseases

MDPI

Supplemental Table S1. Predictors of Liver Enzymes on Regression (Adjusted Model).

	ALT (U/L)		AST (U/L)		AST/ALT Ratio	
	**OR (95%CI)**	***p*-value**	**OR (95%CI)**	***p*-value**	**OR (95%CI)**	***p*-value**
Age	0.99 (0.98, 0.99)	<0.001 **	0.99 (0.99, 1.00)	0.270	1.02 (1.01, 1.02)	<0.001 **
Gender	0.47 (0.42, 0.52)	<0.001 **	0.41 (0.37, 0.46)	<0.001 **	3.65 (3.28, 4.7)	<0.001 **
Race	0.82 (0.81, 0.89)	<0.001 **	0.91 (0.87, 0.96)	<0.001 **	1.18 (1.13, 1.23)	<0.001 **
BMI	1.05 (1.04, 1.07)	<0.001 **	1.02 (1.01, 1.03)	0.005 *	0.92 (0.91, 0.93)	<0.001 **
HTN	1.18 (1.03, 1.36)	0.017 *	1.27 (1.07, 1.50)	<0.001 **	0.95 (0.85, 1.06)	0.367
Uric Acid [a]	1.58 (1.40, 1.77)	<0.001 **	1.60 (1.37, 1.87)	<0.001 **	0.83 (0.73, 0.94)	0.003 *
	**ALP (U/L)**		**GGT (U/L)**		**Total Bilirubin (μmol/L)**	
	**OR (95%CI)**	***p*-value**	**OR (95%CI)**	***p*-value**	**OR (95%CI)**	***p*-value**
Age	1.02 (1.00, 1.02)	<0.001 **	1.00 (1.00, 1.01)	<0.001**	0.99 (0.99, 1.00)	0.290
Gender	1.33 (0.99, 1.80)	0.058	1.70 (1.42, 2.04)	<0.001 **	0.34 (0.30, 0.40)	<0.001 **
Race	0.81 (0.73, 0.90)	<0.001 **	0.95 (0.88, 1.02)	0.171	0.98 (0.93, 1.05)	0.607
BMI	1.04 (1.03, 1.05)	0.026 *	1.04 (1.03, 1.04)	<0.001 **	0.93 (0.92, 0.94)	<0.001 **
HTN	1.39 (1.04, 1.85)	0.026 *	1.43 (1.18, 1.72)	<0.001 **	0.96 (0.81, 1.13)	0.607
Uric Acid [a]	0.82 (0.56, 1.19)	0.282	1.81 (1.52, 2.17)	<0.001 **	1.63 (1.42, 1.88)	<0.001 **

OR = Odds Ratio; CI = Confidence Interval; BMI: Body Mass Index; HTN: Hypertension (135/85 mmHg); Significant at $*p < 0.05$ and $**p < 0.001$. [a] Gender-based cut-off value [(<6.8 (M) <5.7 (F); ≥6.8 (M) ≥5.7 (F)].

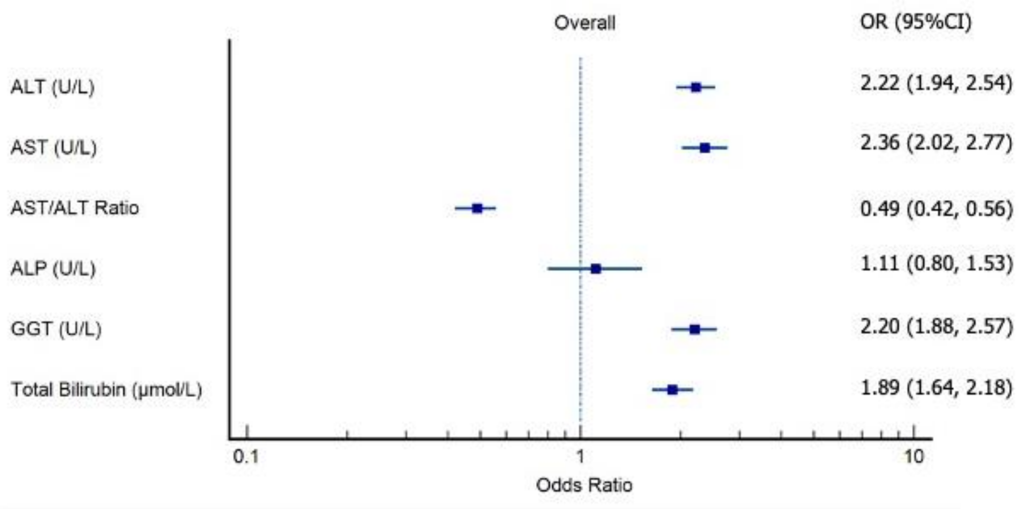

Figure S1. Forest plots of the odds of elevated liver enzymes with hyperuricemia using universal cut-off value.

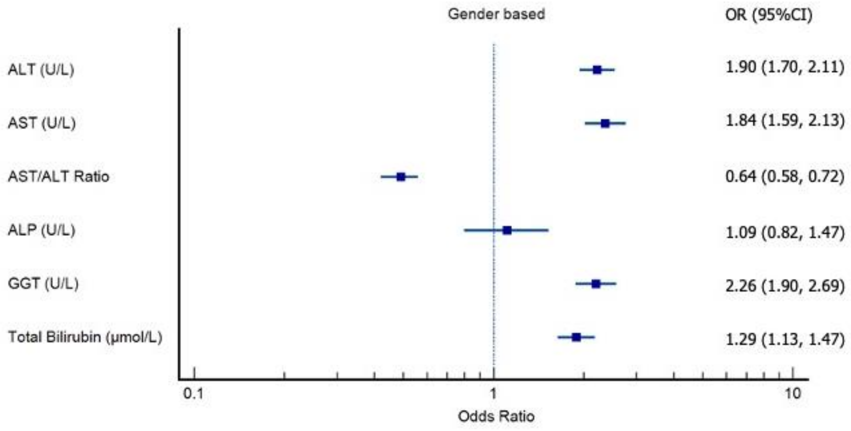

Figure S2. Forest plots of the odds of elevated liver enzymes with hyperuricemia using gender-based cut-off values.
